# Bra strap orientations and designs to minimise bra strap discomfort and pressure during sport and exercise in women with large breasts

**DOI:** 10.1186/s40798-015-0014-z

**Published:** 2015-05-01

**Authors:** Celeste E Coltman, Deirdre E McGhee, Julie R Steele

**Affiliations:** Biomechanics Research Laboratory, University of Wollongong, Northfields Avenue, Wollongong, New South Wales 2522 Australia

## Abstract

**Background:**

Bra straps are a primary source of discomfort during sport and exercise, particularly for women with large breasts. This study aimed to investigate the effects of altering bra strap orientation and design on bra strap comfort, pressure and breast support in women with large breasts. This is a descriptive laboratory study.

**Methods:**

Bra strap discomfort (visual analogue scale, 0 to 10), pressure (custom-designed 10 mm^2^ calibrated pressure sensor, 0.5 to 24 kPa range, 50 Hz, S2011, Novel GmbH, Munich, Germany, placed under the right bra strap at the crest of each participant’s shoulder), preference ranking and vertical breast displacement (VBD; Optotrak Certus® motion capture system, 200 Hz, Northern Digital, Ontario, Canada) data during dynamic treadmill running and static upright standing (pressure only) were collected for 23 active women with large breasts (D+ cup size) while they wore an encapsulation sports bra with six different bra strap conditions (two bra strap orientations: vertical and cross-back; three bra strap designs: standard width, wide and gel).

**Results:**

Bra strap discomfort was significantly less (*p* ≤ 0.001) in the vertical compared to the cross-back strap orientation, which was the most preferred orientation despite no significant difference in strap pressure. The wide strap design had the lowest discomfort scores, significantly lower strap pressure compared to the standard width and gel strap designs (*p* < 0.001), and was equally the most preferred design with the gel straps. There was no significant difference in VBD among the six strap conditions.

**Conclusions:**

Bra straps that are vertically orientated and wide (approximately 4.5 cm in width) are preferable for women with large breasts during sport and exercise to minimise bra strap pressure and discomfort. The addition of gel pads under bra straps may also decrease discomfort and prevent straps slipping off the shoulders, although this notion warrants further investigation.

## Key points

- This study provides evidence upon which sports medicine practitioners can offer advice regarding bra strap orientations and designs most suitable to minimise bra strap discomfort and pressure in active women with large breasts.
- Encapsulation sports bras with bra straps that are vertically orientated and wide (approximately 4.5 cm in width) are preferable for women with large breasts to wear during sport and exercise in order to minimise bra strap pressure and discomfort.
- The addition of gel pads under bra straps may also decrease discomfort and potentially prevent straps slipping off the shoulders.

## Background

Women with large breasts suffer from musculoskeletal pains secondary to their breasts, including head, neck, back and shoulder pain [1,2,3]. Although bras provide external support to the breasts, they also contribute to these musculoskeletal pains experienced by women with large breasts [4]. One bra component that contributes to these pains is the bra straps. Providing secondary breast support to the band of the bra, straps have been rated as the most disliked features of current bra design [5]. The weight of large breasts, which is exerted through the bra straps onto the superior aspect of the shoulders, often over prolonged durations of bra wear, can create deep furrows and soft tissue damage at the bra strap-shoulder interface [6]. In extreme cases, the prolonged pressure and tissue deformation caused by excessive bra strap loading can lead to neurological symptoms in the upper limbs [6]. Reducing this loading through reduction mammoplasty surgery has been found to be effective in relieving bra strap discomfort and neurological symptoms [7]. Breast reduction surgery, however, is not always feasible due to the high financial and personal costs of surgery and the risks of post-surgery complications in women with a body mass index (BMI) greater than 26 [8-11].

One treatment strategy that has been found to decrease the local discomfort and tissue tenderness at the bra strap-shoulder interface is bra removal [4]. Removing the external breast support provided by a bra, however, is likely to have a negative impact on the posture of women with large breasts [1,12]. Research has shown an increased flexion torque on the thoracic spine among women with large breasts, with this torque thought to contribute to the greater thoracic kyphosis found in women with large breasts compared to women with small breasts [1,12]. It is acknowledged that breast mass is also affected by breast density, which varies widely among women [8,13]. Furthermore, bra removal may also have a negative effect on the physical activity level of women with large breasts because breast discomfort and embarrassment related to excessive breast movement are barriers to women participating in sport and exercise [14-16]. Promoting physical activity in women with large breasts is particularly important as breast mass and BMI are positively correlated [17], and it is imperative that all women, irrespective of breast mass, are able to enjoy the health benefits associated with an active lifestyle. Therefore, improving sports bra design is a more sensible treatment strategy to reduce local discomfort and tissue tenderness at the bra strap-shoulder interface compared to bra removal [4].

**Figure 1:**
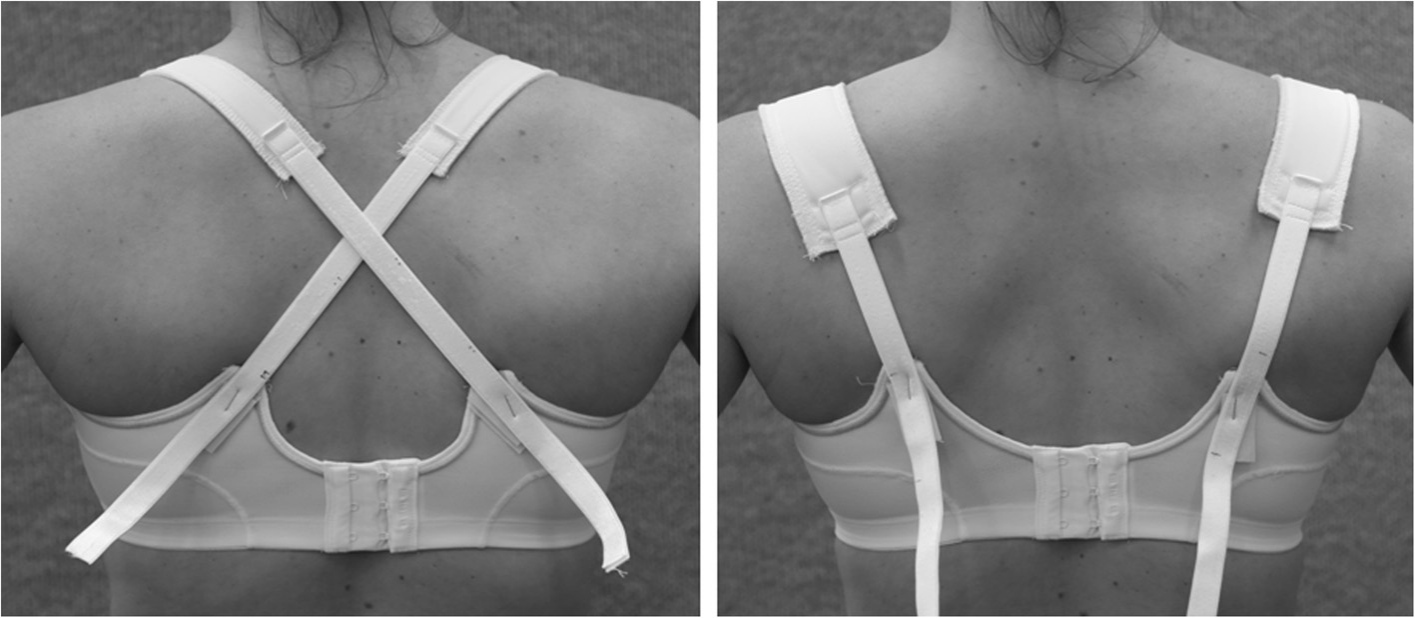
**Strap design.** The standard width strap design in the cross-back orientation (left) and the wide strap design in the vertical strap orientation (right). All bra straps were constructed using the same industrial grade bra wadding (outer layer: 100% polyester; inner layer: 65% polyprople, 35% polyester), cotton spandex (95% cotton, 5% spandex) and satin power mesh (88% nylon, 12% spandex), as typically used in the straps of commercially available encapsulation bras.

Currently, sports bras have several different strap orientations, with some bras being able to convert from one strap orientation to another. The common strap orientations are a vertical orientation, where the straps run vertically over the shoulders, lying on the acromion process and lateral clavicle at the apex of the shoulder, and a cross-back orientation, where the straps cross the back and lie on the upper trapezius muscles at the apex of each shoulder (Figure 1). In terms of design, bra straps also vary in width and the materials the straps are made of. The standard commercially available sports bra strap width at the apex of the shoulder is approximately 2.5 cm (e.g. High Performance Non-Padded Sports Bra, Berlei, Wentworthville, NSW, Australia), although this width can vary. Indeed, the bra strap width of fashion bras can be as narrow as 0.8 cm (Wind Chime Balconette bra, Elle Macpherson Intimates, Sydney, NSW, Australia). Different materials, such as gel pads, are also incorporated into some commercially available sports bra straps (e.g. Triaction Sports bra, Triumph, Bad Zurzach, Switzerland).

Only one previous study has investigated the effect of altering bra strap orientation and design on both bra strap discomfort and pressure [5]. Vertical and cross-back bra strap orientations were compared with and without the addition of a small cushioned pad placed under the strap at the apex of the shoulder. Bra strap discomfort was not significantly different in any condition and bra strap pressure was only reduced with the addition of the cushioned pad in the cross-back orientation, which had higher pressures than in the vertical strap orientation, both with and without the cushioned pad. Although the results of this previous study suggest that bra strap discomfort and pressure cannot be reduced by altering bra strap orientation and design, the study had several limitations. For example, the authors reported that the cushioned pad was not effective in increasing strap width or, in turn, surface area of contact at the shoulder-strap interface as it was found to curl under the strap in the vertical condition [5]. The higher strap pressures in the cross-back orientation may also have been due to tighter straps in this orientation because the bra straps used in the study were commercially available convertible straps, which do not always allow for the greater distance required between the attachment sites of the bra in the cross-back orientation compared to the vertical orientation. It is also conflicting that the cross-back orientation was the most preferred by the 14 participants when it had the same level of discomfort as the vertical orientation and higher strap pressures. The participant number was also low, and the bra sizes, and in turn breast sizes, were at the small end of the spectrum for women with large breast [17-20]. It is also recognised that a range of reasons can affect bra strap preference, such as aesthetics and personal preference, which may have resulted in the cross-back orientation being the preferred orientation in the study cohort.

Due to the limitations listed above, further research is required to determine whether alterations in bra strap orientation and design can decrease bra strap discomfort and pressure in women with large breasts. Such research could provide sports medicine practitioners with evidence regarding which bra strap orientations and designs are most suitable for their female patients with large breasts to wear during sport and exercise to minimise breast movement and discomfort considering age, breast size and level of physical activity [21]. The aim of this study was therefore to investigate the effects of altering bra strap orientation and design on bra strap comfort, pressure and breast support for active women with large breasts. It was hypothesised that both strap orientation and design would affect the pressures exerted at the bra strap-shoulder interface and, in turn, ratings of self-reported bra strap discomfort and preference, without compromising breast support.

## Methods

### Participants

Twenty-three women (22.3 ± 2.6 years of age, 168.5 ± 5.0 cm height, 66.3 ± 6.5 kg body mass) who were professionally fitted [22] to wear a D+ cup bra size (average cup size DD, range D to E; average band size 10, range 10 to 16) were recruited as representative of women with large breasts. The cohort was deliberately homogenous in age range, mass and physical activity levels (all reported to exercise in a sports bra approximately 5 h per week) [23] to eliminate differences due to these variables and to ensure that sports bras were commonly used by the participants. Exclusion criteria included previous breast surgery, currently experiencing menopause, being pregnant or breastfeeding, or suffering from any musculoskeletal disorder or pain that prevented treadmill running. Based on a power analysis using G*power 3.1.3 and that a difference of ±2 on a visual analogue scale (VAS; rated 0 to 10) measuring discomfort was deemed significant [19], it was estimated that a minimum sample size of 22 participants was required to achieve statistical power of at least 80% (with a significance level of *p* < 0.05). To account for potential participant drop out, 23 participants were recruited for the present study. Participants were not tested if they were experiencing any breast tenderness associated with their menstrual cycle. Each participant completed a short questionnaire about their current sports bra usage and provided written informed consent before participating in the study. The University of Wollongong Human Research Ethics Committee (HE12/118) approved recruitment and testing procedures, and all testing was conducted in accordance with the National Statement on Ethical Conduct in Human Research [24].

### Experimental design

A within-subject design was used, where participants ran in a standardised manner and speed (average speed: 9.1 ± 0.3 kph) on a treadmill (PowerJog, GX-100; Expert Fitness UK, Glamorgan, UK) while wearing the same style of encapsulation sports bra (New Legend Underwire sports bra, Berlei, Wentworthville, NSW, Australia), which provided a high level of support and is recommended for women with large breasts to wear during high-impact physical activity [22]. Six randomly [25] allocated bra strap conditions were trialled, including two bra strap orientations (vertical and cross-back) and three different bra strap designs (standard width, wide and gel). All of the straps were made and sewn by the primary investigator [CEC] to ensure a standardised bra strap structure across the three strap designs and a standardised length of the two strap orientations per participant, which were longer in the cross-back orientation compared to the vertical orientation. Professional bra fitting criteria [5,22] were used to ensure the bras fitted the participants correctly and that each strap was the correct length for each participant, in each strap condition (strap length was adjusted once the gel pad was added), as both incorrect bra fit and insufficient strap length could bias both bra strap discomfort and pressure measurements. Following adequate familiarisation, the participants ran in each bra strap condition for 3 min, with data collected while the participants stood motionless (static condition) prior to running and then between the first and third minute of running (dynamic condition). A three-minute duration was chosen as the baseline duration for each condition to minimise participant burden and to facilitate comparison of the strap conditions by limiting the time between them, although it is acknowledged that women commonly exercise and wear sports bras for much longer durations. All the running trials were closely supervised to standardise both the mode of running and the speed, and at least 5 min of rest was allowed between conditions. Participants wore their own running shorts and shoes, which were checked to ensure that they were appropriate for the running task.

### Experimental bra strap conditions

**Figure 2:**
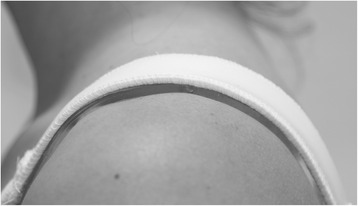
**The Dermis Plus Polymer gel pads.** These were cut into strips and placed under the standard width bra strap design to create the gel strap design. Each gel pad was cut into four equal 10 cm × 2.5 cm strips, which were positioned end to end and then placed under the standard width bra straps, so that the gel material was flush with the bra strap and in direct contact with the participant’s skin.

The standard width and wide strap designs were made of the same material, consistent with commercially available encapsulation sports bra straps (see Figure 1). The gel strap design consisted of the standard width strap, with the addition of a 2.5-cm-wide gel pad (Dermis Plus Polymer gel; MacMed Health Care, Mudgeeraba, Queensland, Australia) placed under the bra strap (see Figure 2). Each strap was secured to the test bra in the same manner, anteriorly with bikini hooks (15 mm × 2 mm white plastic hooks; Birch Haberdashery & Craft, Heidelberg, Victoria, Australia) and posteriorly with hook and loop tape (Birch Haberdashery & Craft, Heidelberg, Victoria, Australia), staples and strapping tape. Each participant was provided with a new test bra, two new sets of bra straps (standard width (2.5 cm) and wide (4.5 cm)) and two unused gel strips for hygiene purposes and to eliminate potential effects of wear or washing on the bra straps.

### Bra strap discomfort and strap preference

Bra strap discomfort was measured immediately after the running trials using a VAS (rated 0 to 10), whereby 0 represented ‘no discomfort’ and 10 represented ‘worst possible discomfort’. The perceived reason for any bra strap discomfort was also reported, and at the end of the test session, participants selected their most and least preferred bra strap orientation and design.

### Bra strap pressure

Strap pressure exerted on the shoulders (bra strap-shoulder interface) was measured while the participants stood stationary and upright prior to running (static condition) and then while they ran on the treadmill (dynamic condition) during each strap condition. Pressure was measured using a custom-designed 10 mm^2^ calibrated pressure sensor (0.5 to 24 kPa range, 50 Hz, S2011, Novel GmbH, Munich, Germany) placed under the right bra strap at the crest of each participant’s shoulder, where the bra strap traversed the shoulder and exerted a downward force on the sensor. The sensors were secured with micropore surgical tape (3M™ Australia, Sydney, NSW, Australia) and zeroed prior to the 10-s of static pressure data collection, the six 10-s samples during the steady state treadmill running and the 10-s static pressure recording once the running was completed. Pliance-x Expert Online software (Version 10.3, Novel GmbH, Munich, Germany) was used to calculate the average static pressure (kPa), and the average dynamic peak pressure (kPa), which was taken as the average of the six 10-s periods per bra strap condition.

### Vertical breast displacement

Vertical breast displacement (VBD; cm) relative to the torso was measured using an Optotrak Certus® motion capture system (200 Hz, Northern Digital, Ontario, Canada) during dynamic treadmill running to determine whether breast motion was consistent among the different strap conditions. Two infrared-emitting diodes (2 mm diameter) were placed on each nipple using double-sided toupee tape (Creative Hair Products, Melbourne, Victoria, Australia), which was placed over micropore surgical tape (3M™ Australia, NSW). A third diode was placed on the sternal notch as a reference point to characterise trunk motion in the vertical plane. Three-dimensional motion of the three markers was recorded during each running trial for six 10-s periods using First Principles software (Version 1.2.2, Northern Digital Inc., Ontario, Canada). The average VBD (minimum from maximum during dynamic treadmill running) relative to the trunk was calculated from a representative 8-s epoch (equivalent to 15 to 20 consecutive breast cycles) for each of the six 10-s data recordings per condition.

### Statistical analysis

Frequencies of the questionnaire responses and the bra strap preference data for each bra strap condition were calculated. After confirming the data were normally distributed, means and standard deviations were calculated for, these data, as well as, bra strap pressure and VBD data for each strap condition. A two-way ANOVA design with two within factors (strap orientation and strap design) was then used to determine whether there were any significant main effects or interactions of strap orientation (vertical, cross-back) or strap design (standard width, wide, gel) on the outcome variables, with Tukey *post hoc* analyses used to determine where any significant difference lay. All statistical procedures were conducted using the Statistical Package for the Social Sciences (Version 15.0; SPSS Inc., Chicago, IL, USA).

## Results

### Bra strap orientation

The bra strap discomfort scores (*p* ≤ 0.001; Table 1) were significantly less in the vertical strap orientation (0.9 ± 1.2 VAS score) compared to the cross-back strap orientation (1.9 ± 2.1 VAS score). Participants reported greater strap tightness, pressure and ‘digging in’ in the cross-back strap orientation, despite the correct bra fit and bra strap length (vertical mean length 28.9 cm, range 24.0 to 33.5 cm; cross-back length 35.5 cm, range 28.0 to 41.0 cm). The vertical strap orientation was the most preferred orientation by 70% of participants with reports of ‘feelings of less pressure on the trapezius muscle’, ‘straps did not pull on their shoulder blades’ and ‘feelings of superior comfort’ compared to the cross-back orientation. This was despite no significant difference in the mean or peak strap pressure between the two strap orientations (*p* = 0.398; Table 2).

**Table 1:** **Mean ± standard deviations for strap discomfort and frequency of most and least preferred conditions (** ***n*** **= 23)**

**Strap condition**	**Bra strap discomfort**	**Most preferred** ***n*** **(%)**	**Least preferred** ***n*** **(%)**
Vertical orientation			
Standard	1.4 ± 1.6	1 (4)	5 (22)
Wide	0.5 ± 0.9	8 (35)	0 (0)
Gel	0.7 ± 1.0	7 (31)	2 (9)
Cross-back orientation			
Standard	2.1 ± 2.1*	1 (4)	8 (35)
Wide	1.8 ± 2.3*	2 (9)	7 (30)
Gel	1.9 ± 1.8*	4 (17)	1 (4)

*Indicates a significant main effect of bra strap orientation (*p* < 0.05).

**Table 2:** **Mean ± standard deviation and confidence interval (CI) values for the mean and peak bra strap pressures (kPa) recorded at the bra strap-shoulder interface for each of the six bra strap conditions (** ***n*** **= 15)** ^**a**^

	**Mean static pressure**			**Mean peak pressure**		
	**Mean ± SD**	**95% CI**		**Mean ± SD**	**95% CI**	
		**Lower bound**	**Upper bound**		**Lower bound**	**Upper bound**
Vertical orientation						
Standard	4.3 ± 1.2*	3.7	4.9	9.1 ± 3.6	7.7	10.5
Wide	3.5 ± 1.2*	2.8	4.1	6.2 ± 2.3*	4.8	7.6
Gel	5.6 ± 1.1*	4.9	6.2	10.6 ± 2.4	9.2	12.0
Cross-back orientation						
Standard	4.9 ± 1.5*	4.3	5.5	9.0 ± 2.5	7.6	10.4
Wide	3.2 ± 1.0*	2.5	3.8	5.7 ± 1.5*	4.3	7.1
Gel	5.8 ± 1.2*	5.2	6.4	9.8 ± 3.4	8.4	11.2

*Indicates a significant main effect of bra strap design (*p* < 0.05). ^a^Due to technical issues, pressure data were available for only 15 of the 23 participants. This reduced the statistical power of the pressure data to 68%.

### Bra strap design

There was no significant difference in the bra strap discomfort scores among the three strap designs, although the wide design had the lowest VAS scores (Table 1). Strap preference was similar for the gel and wide designs (gel *n* = 11, 48%; wide *n* = 10, 43%), and the standard width strap was the least preferred design (*n* = 13, 57%; Table 1). When the data were pooled across designs, both the mean static and the dynamic mean peak bra strap pressures were significantly lower in the wide strap design compared to both the standard width and the gel designs (*p* < 0.001). There was no difference in dynamic mean peak pressure between the standard and gel designs (*p* > 0.05; Table 2), although there was a significantly greater mean static pressure in the gel design compared to the standard width design (*p* < 0.001; Table 2). There was no significant difference in VBD among any of the six bra strap conditions (Table 3). Participants’ responses to the questionnaire are provided in Table 4.

**Table 3:** **Mean ± standard deviation values for right vertical breast displacement (cm) during treadmill running (** ***n*** **= 23)**

**Strap condition**	**Vertical breast displacement (cm)**
Vertical orientation	
Standard	3.3 ± 1.0
Wide	3.2 ± 1.1
Gel	3.3 ± 1.1
Cross-back orientation	
Standard	3.1 ± 1.1
Wide	3.1 ± 1.0
Gel	3.2 ± 1.1

**Table 4:** **Questionnaire responses for the participants (** ***n*** **= 23)**

**Questionnaire response**	**Number**	**Percentage (%)**
Bra strap orientation of own sports bra		
Vertical	12	52
Cross-back	11	48
Report problems specifically associated with bra straps	15	65
Change bra strap orientation based on clothing worn over bra	8	35
Sacrifice comfort for this change in bra strap orientation	14	61
Report difficulty finding a good sports bra	20	87
Commonly wear more than one bra during exercise	12	52
Have never been professionally fitted for a sports bra	20	87

## Discussion

Sports medicine practitioners should routinely include breast support assessment and education as an integral part of treating active women, particularly those who experience high frequencies and long durations of breast bounce, or to alleviate the musculoskeletal pains suffered by women with large breasts so they can exercise in comfort [12,26,27]. This study provides evidence for sports medicine practitioners upon which to base recommendations on the bra strap orientation and designs most suitable for their female patients, particularly those with large breasts. As breast support should not be compromised for the sake of greater bra strap comfort, the current study ensured the level of breast support was standardised among the six strap conditions, with no significant between-strap condition difference found in VBD (Table 3).

### Bra strap orientation

A vertical bra strap orientation appears to be more suitable for women with large breasts due to the significantly lower strap discomfort and the preference for this orientation compared to the cross-back strap orientation. Although the mean static and dynamic mean peak pressure data were not significantly lower in the vertical bra strap orientation compared to the cross-back orientation, participants consistently reported that the vertical orientation did not ‘dig in’ or ‘create pressure or tension on the trapezius muscle’ compared to the cross-back orientation. It is possible that this between-strap orientation difference in perception of pressure, despite the lack of any quantitative difference in strap pressure, may be due to variations in the anatomical structures at the strap/pressure sensor interface. That is, in the vertical orientation, the strap lays across the bone, which is better designed to tolerate compressive forces relative to the muscle tissue at the cross-back orientation strap/pressure sensor interface (upper trapezius muscle) [28]. As the effect of different tissue interfaces on pressure measurements is unknown, this is a recommended topic for future research.

The lack of difference in strap pressure in the two strap orientation conditions was in contrast to the previous study of Bowles and Steele [5] that reported higher strap pressures in cross-back orientated straps compared to vertical straps [5]. The mean peak pressures were of a lower range in the current study compared to those reported by Bowles and Steele [5] (current study 5.7 to 10.6 versus 11.7 to 14.9 kPa) [5], which is likely to be attributed to differences in the measurement devices (current study: one 10 mm^2^ sensor versus ten 1 cm^2^ sensors positioned in parallel) [5]. Ideally, it would be preferable to have the pressure sensors the same width as the bra strap. However, it is also possible that pressures in the cross-back orientation measured by Bowles and Steele [5] were falsely high due to overly tight straps. That is, the straps used by Bowles and Steele [5] were commercially available convertible bra straps of a fixed length, which did not allow for the additional length required to traverse the torso in the cross-back orientation. Tighter straps will cause higher strap pressures and more strap discomfort. An objective measure of strap tension would provide further insight into the strap pressure/discomfort relationship and is a suggested area of study for future research. Irrespective of differences in strap tension, it is imperative that both bra manufacturers and consumers ensure that the strap length is sufficient for bras with a cross-back strap orientation, taking into consideration the wide range of women’s torso morphology.

The significantly lower strap discomfort and the strap preference of the vertical orientation compared to the cross-back orientation were also in contrast to previous research [5]. It should be noted, however, that the bra strap discomfort VAS scores for both studies were low, confirming that 3 to 5 min of treadmill running is insufficient to assess the long-term impact of bra strap discomfort; as for this cohort, 63% reported discomfort and problems with the bra straps of their own bras (Tables 1 and 4). Future research should therefore ensure that the duration over which bra strap discomfort and pressure are measured are longer to truly represent times that women wear sports bras while they participate in sport and exercise. Other factors may have also contributed to strap preference, such as the clothing to be worn on top of the bra, whether it was acceptable for the bra straps to be visible or not, as well as personal preference. We attempted to control these factors in the current study by assessing personal preference and by recruiting an equal percentage of the participants who reported to commonly wear either strap orientation. Interestingly, 80% of the participants stated that they sacrificed comfort for appearance when changing their bra strap orientation to suit the clothing to be worn over the bra (Table 4).

### Bra strap design

A wide strap design, approximately 4.5 cm in width, is more suitable for women with large breasts compared to the standard width (2.5 cm) or a gel strap design for sports bras, as evidenced by the strap preference and the bra strap pressure data. Participants consistently perceived that the wide straps better cushioned the load borne by the straps, which is logical considering the greater surface area over which to distribute the load generated at the bra strap-shoulder interface. This was also evident in the significantly lower mean static and dynamic mean peak pressure recordings in the wider bra strap conditions compared to the other strap conditions [29]. However, although the wide strap design had the lowest VAS scores, no significant difference was found in strap discomfort among the three strap designs, which may also be attributed to the short durations over which the straps were worn.

The gel strap design had some interesting results that warrant further investigation in future research, in particular combining the gel and the wide strap design. The gel design had equivalent strap discomfort and preference compared to the wide strap design, with participants consistently reporting that they ‘liked’ the feeling of the gel material on their shoulders and felt it ‘cushioned’ the load borne by the straps. This was despite the gel design having the highest mean bra strap pressure. This apparently conflicting result might have been due to limitations in the pressure measurement device used in the current study. That is, only one sensor was placed at the apex of the shoulder, which in the gel condition may have masked the true effect of the gel pad by limiting the surface area that the gel covered, as the gel pad was placed under the standard width bra strap. Furthermore, pressure data were only extracted for 15 of the 23 participants. Future research should measure bra strap pressure using several pressure sensors placed along the entire length of the bra strap or incorporate the gel within the bra strap. The tacky nature of the gel may also have the potential to decrease strap slippage, a problem reported by 57% of women [30], and which also warrants further investigation.

It is common for women with large breasts to experience tissue deformation at the bra strap-shoulder interface, with deep grooves and tissue tenderness caused by their bra straps. The magnitude of bra strap pressures that may cause this deformation, over the long durations (12 to 14 h/day, for up to 60 or 70 years) that women wear bras for, is unknown. Back-pack strap studies have reported pressures as low as 9 kPa caused by carrying 20-kg back-packs for 2 h can produce skin and subcutaneous soft tissue damage in animal studies [31], as well as cause restrictions in circulation within the upper trapezius muscle [32-34]. The bra strap pressures in the current study were mostly below this magnitude (mean static: 3.2 to 5.8 kPa; mean peak dynamic: 5.7 to 10.6 kPa). When considered, however, from an accumulation perspective, the static bra strap pressures are typically sustained for 12 to 14 h a day over many years with the higher dynamic pressures experienced every time a woman participates in sport and exercise. The magnitude of the mean peak bra strap pressures during the running trials also suggest that the frequency of breast bounce should be considered when advising active women on breast support, regardless of their breast size. That is, the breasts bounce with each heel strike. Therefore, if active women are involved in hours per week of impact activity, which could equate to tens of thousands of breast bounces per week, they should ensure that the bras they wear have straps that are designed to minimise strap pressure in order to reduce development of tissue tenderness and deformation at the bra strap-shoulder interface. Further research is required to investigate the effects of longer durations and higher frequencies of dynamic bra strap pressures on tissue loading and circulation within the upper trapezius muscles in women with a range of breast sizes [31,32].

## Conclusions

The current study provides evidence upon which sports medicine practitioners can offer advice regarding bra strap orientations and designs most suitable to minimise bra strap discomfort and pressure in active women, particularly those women with large breasts. Encapsulation sports bras with bra straps that are vertically orientated and wide (approximately 4.5 cm in width) are preferable for women with large breasts to wear during sport and exercise in order to minimise bra strap pressure and discomfort. The addition of gel pads under the bra strap may also decrease discomfort and potentially prevent the strap slipping off the shoulders, although this notion warrants further investigation.
